# Supplementary material for: Single-amino acid variants reveal evolutionary processes that shape the biogeography of a global SAR11 subclade
Source: eLife. 2019 Sep 3;8:e46497. doi: 10.7554/eLife.46497 (PMC6721796; doi:10.7554/eLife.46497)
Supplement: Transparent reporting form [file elife-46497-transrepform.docx]

***eLife’s* transparent reporting form**

We encourage authors to provide detailed information *within their submission* to facilitate the interpretation and replication of experiments. Authors can upload supporting documentation to indicate the use of appropriate reporting guidelines for health-related research (see [EQUATOR Network](http://www.equator-network.org/%20)), life science research (see the [BioSharing Information Resource](https://biosharing.org/)), or the [ARRIVE guidelines](http://www.plosbiology.org/article/info:doi/10.1371/journal.pbio.1000412) for reporting work involving animal research. Where applicable, authors should refer to any relevant reporting standards documents in this form.

If you have any questions, please consult our Journal Policies and/or contact us: [editorial@elifesciences.org](mailto:editorial@elifesciences.org).

**Sample-size estimation**

- You should state whether an appropriate sample size was computed when the study was being designed
- You should state the statistical method of sample size computation and any required assumptions
- If no explicit power analysis was used, you should describe how you decided what sample (replicate) size (number) to use

Please outline where this information can be found within the submission (e.g., sections or figure legends), or explain why this information doesn’t apply to your submission:

Our analysis of surface ocean metagenomes to characterize environmental genomic heterogeneity of closely related SAR11 populations makes use of the Tara Oceans Project metagenomes (n=93) that have been made publicly available by Sunagawa et al. (2015), and arctic metagenomes (n=10) from the Ocean Sampling Day Project (Kopf et al., 2015) to complement missing arctic samples from the Tara Oceans Project. We haven’t performed an explicit power analysis, however, we employed all available surface ocean metagenomes in the Tara Oceans Project for our metagenomic read recruitment analyses.

**Replicates**

- You should report how often each experiment was performed
- You should include a definition of biological versus technical replication
- The data obtained should be provided and sufficient information should be provided to indicate the number of independent biological and/or technical replicates
- If you encountered any outliers, you should describe how these were handled
- Criteria for exclusion/inclusion of data should be clearly stated
- High-throughput sequence data should be uploaded before submission, with a private link for reviewers provided (these are available from both GEO and ArrayExpress)

Please outline where this information can be found within the submission (e.g., sections or figure legends), or explain why this information doesn’t apply to your submission:

We removed metagenomes in which the SAR11 population was covered less than 50X. They were outliers in an ecological sense rather than a statistical sense: our microbial population of interest was not abundant to analyze its genomic heterogeneity in these metagenomes confidently.

**Statistical reporting**

- Statistical analysis methods should be described and justified
- Raw data should be presented in figures whenever informative to do so (typically when N per group is less than 10)
- For each experiment, you should identify the statistical tests used, exact values of N, definitions of center, methods of multiple test correction, and dispersion and precision measures (e.g., mean, median, SD, SEM, confidence intervals; and, for the major substantive results, a measure of effect size (e.g., Pearson's r, Cohen's d)
- Report exact p-values wherever possible alongside the summary statistics and 95% confidence intervals. These should be reported for all key questions and not only when the p-value is less than 0.05.

Please outline where this information can be found within the submission (e.g., sections or figure legends), or explain why this information doesn’t apply to your submission:

The most critical statistical approach our study made use of is a graph-based activity regularization technique for competitive learning from hyper-dimensional data. While we justified the use and details of this statistical approach with two citations, we also made available an extensive description of the workflow in our supplementary data, and specific parameters online (<http://merenlab.org/data/2018_Delmont_et_al_SAR11_SAAVs/)> to ensure full transparency and reproducibility.

Details of all other statistical tests are clearly written in our Results and Methods sections.

(For large datasets, or papers with a very large number of statistical tests, you may upload a single table file with tests, Ns, etc., with reference to sections in the manuscript.)

**Group allocation**

- Indicate how samples were allocated into experimental groups (in the case of clinical studies, please specify allocation to treatment method); if randomization was used, please also state if restricted randomization was applied
- Indicate if masking was used during group allocation, data collection and/or data analysis

Please outline where this information can be found within the submission (e.g., sections or figure legends), or explain why this information doesn’t apply to your submission:

Our study does not make use of an experimental grouping of input metagenomes.

**Additional data files (“source data”)**

- We encourage you to upload relevant additional data files, such as numerical data that are represented as a graph in a figure, or as a summary table
- Where provided, these should be in the most useful format, and they can be uploaded as “Source data” files linked to a main figure or table
- Include model definition files including the full list of parameters used
- Include code used for data analysis (e.g., R, MatLab)
- Avoid stating that data files are “available upon request”

Please indicate the figures or tables for which source data files have been provided:

All raw, intermediate, and final data are extensively reported in our study through supplementary tables and data packs associated with digital object identifiers, along with a fully reproducible bioinformatics workflow for full transparency.
